# Determinants of change in blood pressure in Ghana: Longitudinal data from WHO-SAGE Waves 1–3

**DOI:** 10.1371/journal.pone.0244807

**Published:** 2021-01-08

**Authors:** Elias K. Menyanu, Barbara Corso, Nadia Minicuci, Ilaria Rocco, Joanna C. Russell, Lisa J. Ware, Glory Chidumwa, Nirmala N. Naidoo, Richard B. Biritwum, Paul R. Kowal, Aletta E. Schutte, Karen E. Charlton

**Affiliations:** 1 School of Medicine, Faculty of Science, Medicine and Health, University of Wollongong, Wollongong, New South Wales, Australia; 2 Neuroscience Institute, National Research Council, Padova, Italy; 3 School of Health and Society, Faculty of Social Sciences, University of Wollongong, Wollongong, New South Wales, Australia; 4 SAMRC/Wits Developmental Pathways for Health Research Unit, University of the Witwatersrand, Johannesburg, South Africa; 5 Department of Epidemiology and Biostatistics, School of Public Health, Faculty of Health Sciences, University of the Witwatersrand, Johannesburg, South Africa; 6 World Health Organization Data, Analytics and Delivery for Impact Division, Geneva, Switzerland; 7 University of Ghana, Accra, Ghana; 8 Chiang Mai University Research Institute for Health Sciences, Chiang Mai, Thailand; 9 World Health Organization SAGE, Geneva, Switzerland; 10 School of Public Health and Community Medicine, The George Institute for Global Health, University of New South Wales, Sydney, New South Wales, Australia; 11 Illawarra Health and Medical Research Institute, Wollongong, New South Wales, Australia; University of Mississippi Medical Center, UNITED STATES

## Abstract

The prevalence of hypertension is increasing in low- and middle-income countries, however statistics are generally derived from cross sectional surveys that utilize different methodologies and population samples. We investigated blood pressure (BP) changes over 11–12 years in a large cohort of adults aged 50 years and older (n = 820) included in the World Health Organization’s Study on global AGEing and adult health (WHO-SAGE Ghana) Wave 1 (2007/8) with follow up in Wave 3 (2019). Participants’ BP were measured in triplicate and a survey completed at both time points. Survey instruments collected information on sociodemographic characteristics, lifestyle, health behaviors and chronic conditions. While no significant difference was found in systolic BP between Waves 1 and 3, diastolic BP decreased by 9.7mmHg (mean = 88.6, 15.4 to 78.9, 13.6 respectively) and pulse pressure increased by 9.5mmHg (44.8, 13.7 to 54.3, 14.1). Awareness of hypertension increased by 37%, from (20% to 57%), but no differences were found for the proportion of hypertensives receiving treatment nor those that had controlled BP. Mixed effects modelling showed a decrease in diastolic BP was associated with increasing age, living in rural areas and having health insurance. Factors associated with an increased awareness of hypertension were residing in urban areas, having health insurance and increasing body mass index. While diagnosis of hypertension has improved over time in Ghana, there is an ongoing need to improve its treatment in older adults.

## Introduction

An urgent need to reduce population level blood pressure (BP) is recognized globally by health agencies and governments in order to minimize the associated outcomes of stroke, myocardial infarction, cardiac failure, dementia, renal failure, blindness and premature death [1–9]). A 25% relative reduction in the prevalence of raised BP by 2025 is one of the voluntary global targets of the World Health Organization (WHO) for prevention of non-communicable diseases (NCDs), and resources have been developed to assist countries to achieve this target [9, 10]. However, progress towards this target has been slow, with hypertension remaining a major health challenge in many countries [9, 11–14].

Worldwide, population level BP has been declining particularly for high and middle-income regions while it has remained stable or has increased among low-income countries. In an extensive analysis of 1479 national, subnational and community studies conducted between 1975 and 2015 in 19·1 million participants, significant reductions in mean systolic blood pressure (SBP) and diastolic blood pressure (DBP) over time were demonstrated [15]. In a worldwide study, SBP decreased by 0.8 mmHg and 1 mmHg per decade for males and females, respectively, between 1980 and 2008 with substantial reductions reported in Australia, Western Europe and North America [16]. However, increased SBP has been recorded in the Oceania, east Africa, and south and southeast Asia for both sexes, and in West Africa for women, ranging from an increase of 0·8–1·6 mmHg in men and 1·0–2·7 mmHg in women per decade [16]. Furthermore, studies examining BP across the life-course indicate that, while SBP typically increases continuously until age 70 or 80years, DBP typically rises less steeply than SBP and remains constant or even declines after the fifth to sixth decade of life [17–21]. Consequently, an increased pulse pressure (PP) poses a major cardiovascular risk [19, 22–24]. However, it remains unclear how changes with age in older adults in regions; such as Sub-Saharan Africa (SSA) where population level BP is not declining. With high hypertension rates and low levels of awareness, treatment and control in the African region [25–29], understanding changes over time in older adults is essential in order to reduce associated morbidity and mortality.

To better understand the trajectory of hypertension and predictors of change in BP over time, we aimed to identify changes in population level BP in a nationally representative longitudinal cohort of Ghanaians over an 11–12year period.

## Materials and methods

**Fig 1:**
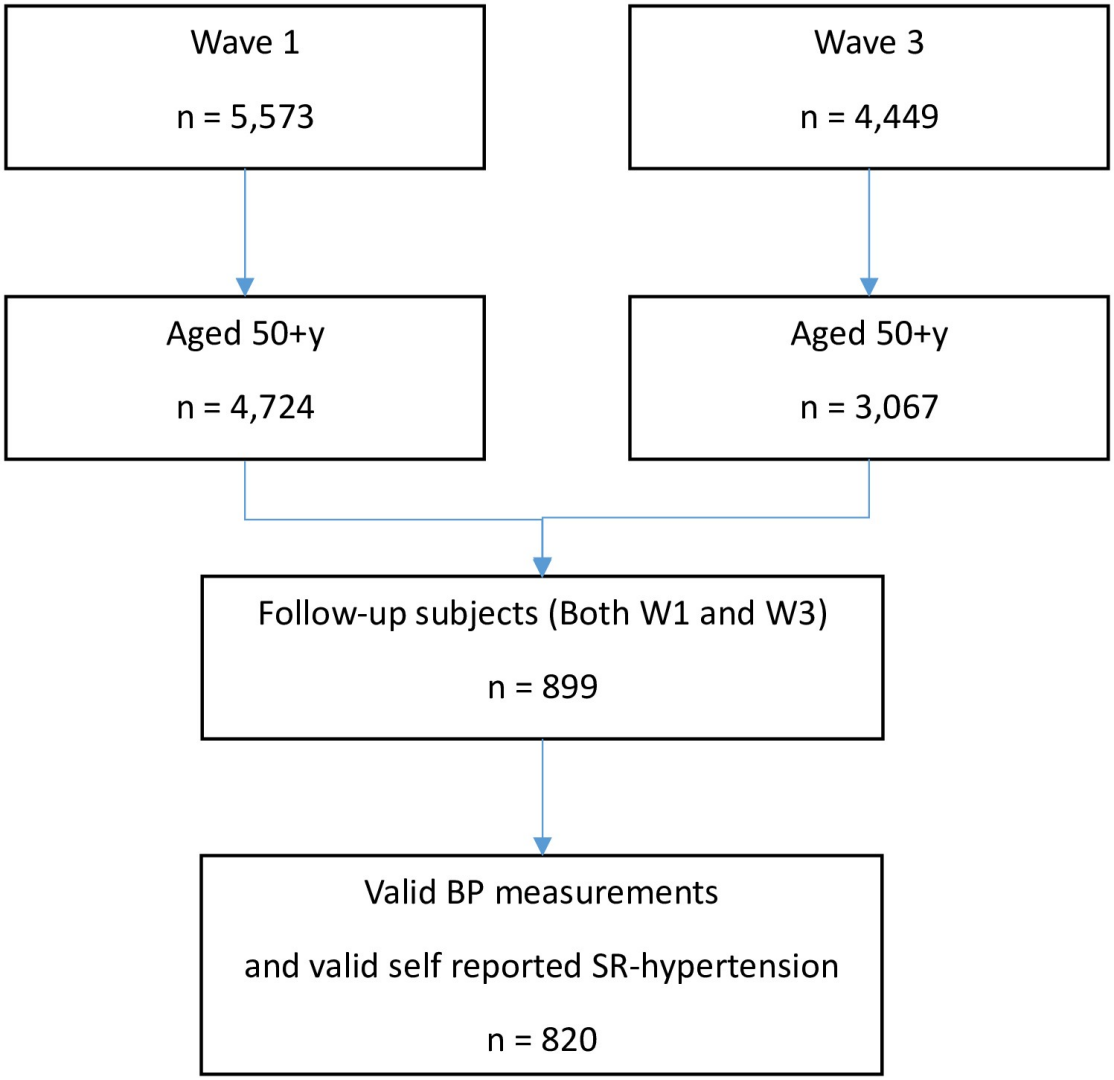
Flow diagram of recruitment and inclusion criteria.

The study utilized a nationally representative sample of Ghanaians who participated in the World Health Organization’s Study on global AGEing and adult health (WHO-SAGE); a population based longitudinal survey conducted in six low and middle-income countries (LMICs) (China, Ghana, India, Mexico, Russia and South Africa) with the aim to provide support through policy, planning and research [30]. For the purpose of this analysis, data from participants 50y and above, included in WHO-SAGE Wave 1 [W1] (2007/2008), were followed up in a preliminary analysis of data from Wave 3 [W3] (2019). The Census Enumerated Areas (CEA) of the 2000 Population and Housing Census was used as the sampling frame. The study design randomly selected 250 enumeration areas (EAs) as the primary sampling units (PSU), nationwide, resulting in 20 strata. The number of EAs to be selected from each strata was based on proportional allocation (determined by the number of EAs in each strata specified on the census frame). In each selected EA, a listing of the households was conducted to classify each household into the following mutually exclusive categories: 1) World Health Survey (WHS)/SAGE Wave 0 follow-up households with one or more members aged 50 years or more; 2) new households with one or more members aged 50 years or more; 3) WHS/SAGE Wave 0 follow-up households which did not include any members aged 50y or more, but included residents aged 18–49y; and, 4) new households which did not include any members aged 50y or more, but included residents aged 18–49y. Within each EA, 24 households were randomly selected (twenty 50+y households and four 18–49y households). In the 50+y households, all participants 50y and above were selected whereas in 18–49y households only one member was selected, details of which are described elsewhere [31]. At each wave of the study, replacements for losses to the sample were included. We followed up 820 participants with valid BP and self-reported hypertension status data from W1 to W3, as shown in Fig 1. We compared the sociodemographic and health characteristics of the 820 subjects (50+ y olds) included in this work with both the n = 3,904 participants who were excluded (i.e. not followed-up and no valid BP data; n = 4,724–820) and the 79 (i.e. n = 899–820, aged 50+y) subjects who were followed-up but had no valid BP data (See S2 and S3 Tables; all the frequencies were unweighted).

### Data collection

While data collection was completed by individual field workers using face-to-face hard copy questionnaires in W1, a computer assisted personal interview (CAPI) approach was utilized in W3 in which field workers, in groups, collected data in assigned EAs. The survey questionnaire included participants’ sociodemographic characteristics, information of lifestyle-related risk factors and preventive health behaviours, and diagnosis and management of chronic conditions. Participant’s anthropometric data were recorded. Interviews were conducted in the home language of the participants. All field workers received one week of training prior to the implementation of each survey, with support from the WHO-SAGE team, using standardized training and survey materials [32]. In both waves, field workers visited participants in their houses and places of work to conduct interviews. Data collection in each wave was continuous and took approximately a year to complete.

### Study measures

The main study outcome for this analysis was BP, which was measured using validated wrist-worn BP devices with positional sensors (Omron R6, Kyoto, Japan) [33]. Following 5 minutes of seated rest, three BP readings were recorded on the left wrist (one-minute between each measurement) while the participant sat with legs uncrossed and the wrist positioned precisely at the level of the heart. An average of the second and third readings were used to determine BP. Hypertension classification was made according to the European Society for Hypertension Guidelines (2018), namely systolic ≥140 and/or diastolic ≥90 mmHg [34]. Hypertension status was measured as self-reported treatment or having a measured BP ≥ 140/90mmHg, while hypertension awareness was based on self-reported previous diagnosis of hypertension in those with BP ≥ 140/90mmHg. Hypertension treatment was determined as self-reported medication use for hypertension in at least two weeks prior to data collection. Hypertension control was assessed as those who self-reported antihypertensive medication use within the last two weeks and had a BP measurement of less than 140/90 mmHg. PP was measured as difference between SBP and DBP, representing the force that the heart generates each time it contracts. Regarding national health insurance status, ‘voluntary’ contributors were defined as those individuals who were not captured by the health insurance scheme as public or civil service workers, while ‘mandatory’ contributors were employees from the public, civil and private sectors. “Current alcohol use” was recorded as “having consumed alcohol in the last 30 days”. Physical activity was measured using the Global Physical Activity Questionnaire [35]. Body mass index (BMI) was calculated as weight (kg) / height (m)^2^ and classified according to recommendations from WHO [36]. Waist to circumference was measured using a flexible tap measure wrapped around the midpoint of the last palpable rib and the top of the hip bone of the participant, ensuring the tape is wrapped over the same spot on the opposite side while the participant is standing with feet together and arms at the sides [37]. Waist to height ratio (WtHR) was then calculated.

Prior to taking part in the study, study measures were explained to participants in their home languages and a written informed consent was obtained for each wave. The study complied with the Declaration of Helsinki with ethical approval from the WHO Ethics Committee (RPC 149) and the University of Ghana Medical School Ethics and Protocol Review Committee (MS-Et/M.03—P 3.1/2005-2006).

### Statistical data analysis

Data were analyzed using Stata Statistical Software: Release 16 (Stata Corp LLC, 2019; College Station, USA). Due to non-responses for some survey items, the number of responses (n) for each variable are included in the tables. The WHO-SAGE Ghana participants are allocated a unique 10-digit identifier code that facilitates linking of participant’s information over the two waves of the study. Categorical variables were compared using Chi square test while the Wilcoxon Signed Ranked Test was used to compare continuous variables in the cohort over time. Change in BP was calculated by subtracting the BP measurement obtained in WHO SAGE W1 from that in WHO-SAGE W3. Nonparametric kernel-density plots were constructed with the kdensity command and the default Epanechnikov kernel function utilized. A kernel density plot is a smoothed representation of a histogram with the area under the curve representing the proportion of values compared to all values and sums to 1. Kernel density shows the probability density estimates of the distribution. Mixed model effects regression modelling was used to assess potential predictors of the longitudinal changes in SBP and DBP between W1 and W3. This statistical method for repeated measurements accounts for correlations among measurements within an individual and variations across participants [38, 39]. Logistic regression was used to access factors associated with change in hypertension awareness.

## Results

Of the total W1 sample (n = 5,573), 899 participants were aged 50y+ and were followed-up in W3. Of these, 820 respondents had complete data on BP and self-reported hypertension diagnosis. Of the W1 sample (n = 5,110), 820 participants aged 50yrs+, had complete BP data at both W1 (median age, 59years) and W3 (median age, 71years) and were included in the analysis. The sample comprised 433 (52.8%) men and 387 women (47.2%), with 364 (44.4%) and 456 (56.6%) living in urban and rural areas, respectively.

We further examined sociodemographic and cardiovascular related risk factors and found several changes. There was an increase in the proportion of participants reporting ever attended school which may reflect participation in adult learning literacy programmes in Ghana [40]. WtHR, BMI, diagnosis of diabetes and having voluntary health insurance increased in the cohort while current alcohol intake and overall physical activity levels decreased significantly between W1 and W3 (Table 1).

**Table 1:** Characteristics of participants in WHO-SAGE, Ghana W1 and W3 (n = 820).

Characteristic	Wave 1	Wave 3	P
Age in years	n = 820	n = 820	<0.01
	59 (13)	71 (13)	
Ever schooled, n (%)	n = 817	n = 820	< 0.001
	419 (51.3)	477 (58.2)	
Years educated for those who have ‘ever schooled’	n = 390	n = 427	< 0.001
	10 (4)	10 (7)	
Marital status:	n = 820	n = 820	< 0.001
Married/ cohabiting, n (%)	489 (59.9)	419 (51.1)	
Waist to height ratio (WtHR)	n = 815	n = 747	< 0.001
	0.51 (0.1)	0.53 (0.1)	
BMI	n = 815	n = 747	< 0.001
	22.2 (5.9)	23.2 (7.1)	
*Current alcohol use (%)*	n = 820	n = 820	
Never	376 (45.9)	452 (51.1)	< 0.001
Current use	240 (29.3)	133 (16.2)	
Yes, but not current	201 (24.5)	235 (28.7)	
Overall Physical Activity, n (%)	n = 812	n = 820	< 0.014
	701 (86.3)	294 (53.9)	
Diabetes, n (%)	n = 820	n = 820	0.001
	17 (2.1)	65 (7.9)	
Depression, n (%)	n = 820	n = 820	0.645
	13 (1.6)	13 (1.6)	
*Health Insurance*, *Yes n (%)*	n = 820	n = 820	< 0.001
	307 (37.4)	706 (86.1)	
SBP, mmHg	n = 820	n = 820	0.783
	131.0 (30.0)	130.5 (27.4)	
DBP, mmHg	n = 820	n = 820	< 0.001
	88.6 (20.5)	78.9 (18.0)	
Pulse Pressure, mmHg	n = 82043.0 (16)	n = 82052.5 (17)	< 0.001
Hypertension	n = 820	n = 820	< 0.001
prevalence, n (%)	426 (52.0)	400 (48.8)	
Hypertension awareness (% hypertension prevalence)	85 (20.0)	228 (57.0)	< 0.001
Hypertension treatment (% hypertension awareness)	66 (77.6)	187 (82.0)	0.101
Hypertension control (% hypertension treatment)	18 (27.3)	99 (52.9)	0.195

All data are presented as median (Interquartile range, IQR) unless otherwise indicated. Whereas walking/cycling refers to such activities completed in a typical week, overall physical activity represents all activities including vigorous, moderate, walking/cycling, vigorous fitness and moderate fitness completed in a typical week. Hypertension prevalence refers to BP ≥140/90 or self-reported treatment while hypertension awareness refers self-reported diagnosis. Hypertension treatment denotes blood pressure medication use (within the last 2 weeks) prior to the survey and hypertension control represents medication use with a measured BP less than 140/90 mmHg. Continuous variables were compared using Wilcoxon signed-rank test; categorical variables were compared using the Chi-Square test.

### Blood pressure changes within the population

**Fig 2:**
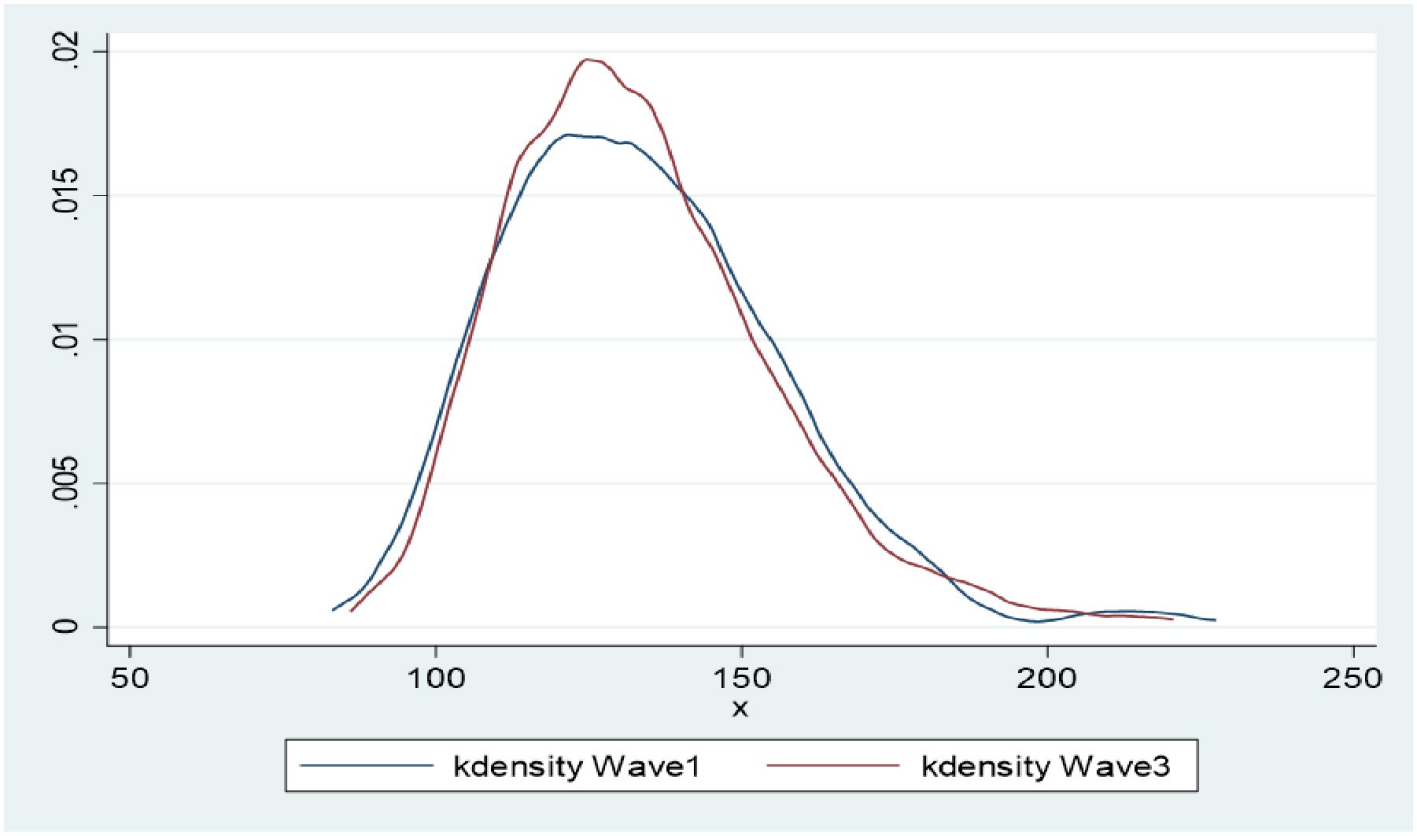
Non-parametric kernel-density estimates for the distribution of SBP (W1, W3 n = 820).

**Fig 3:**
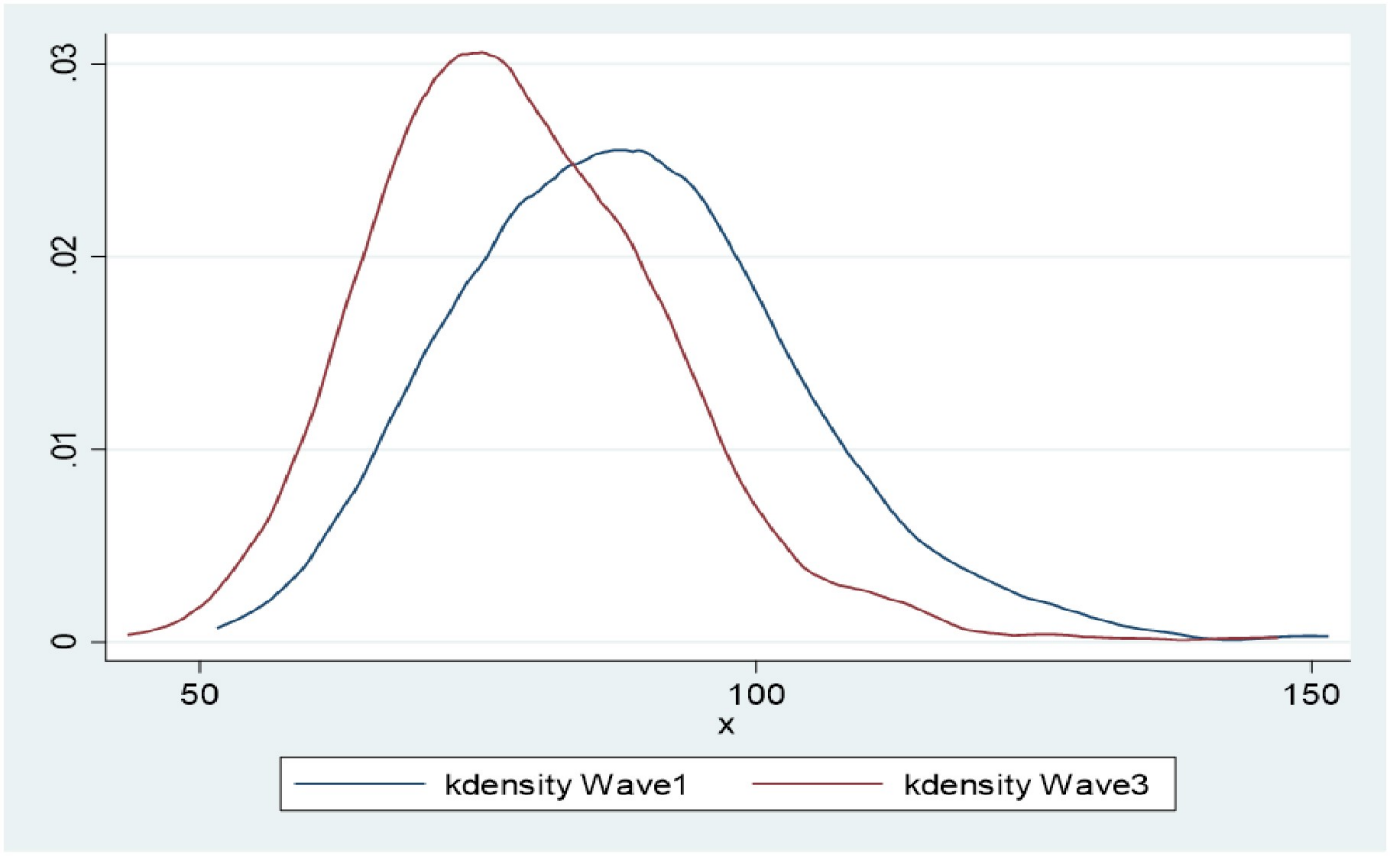
Non-parametric kernel-density estimates for the distribution of DBP (W1, W3 n = 820).

There was no difference in SBP between W1 and W3, however, a significant reduction of 9.7mmHg W1, mean (SD) = 88.6 (15.4); W3, 78.9 (13.6) in DBP was observed over the 11–12 year follow up period. PP significantly increased by 9.4mmHg (95% CI, W1 = 43.9–45.8; W3 = 53.3–55.2). Figs 2 and 3 graphically demonstrate the distributions of SBP and DBP in both waves. While the curves are almost overlapping for SBP, the W3 distribution for DBP is clearly shifted to the left compared to W1.

**Fig 4:**
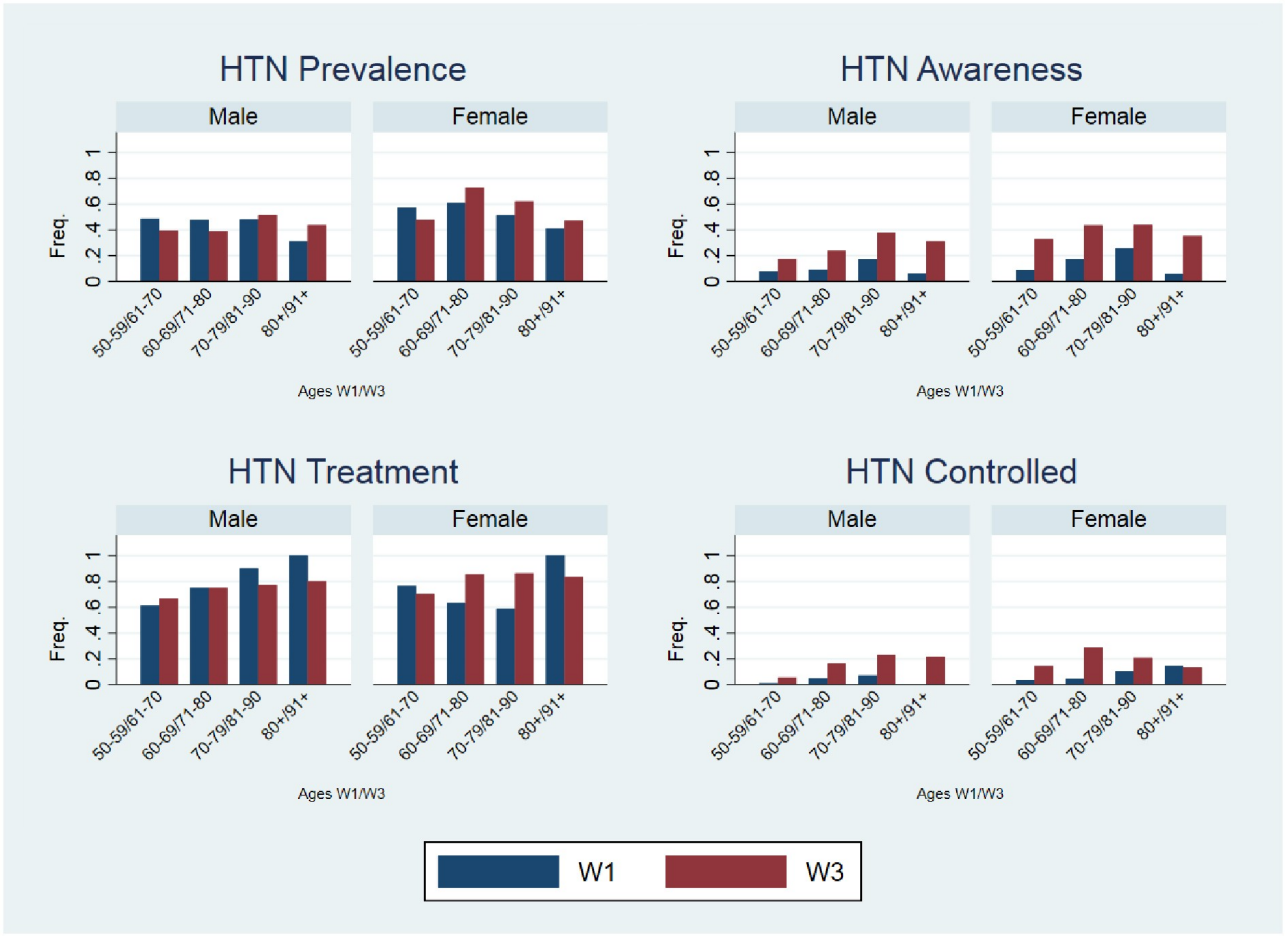
Top left hypertension prevalence (W1 n = 426, W3 n = 400), top right hypertension awareness (W1 n = 95, W3 n = 245), bottom left hypertension treatment (W1 n = 66, W3 n = 187), and bottom right hypertension control (W1 n = 18, W3 n = 99).

Hypertension prevalence decreased significantly by 3.2% over the period (W1, 52.0%, CI = 0.48–0.55; W3, 48.8%, CI = 0.45–0.52). Hypertension awareness increased by 37.0% (p < 0.01) over time with no differences observed for hypertension treatment nor the proportion of treated hypertensive participants that had controlled BP (Table 1). Fig 4 illustrates the distribution of hypertension prevalence, awareness, treatment and control in both waves by sex and age. There were significantly more women than men with hypertension in both waves. Hypertension prevalence was highest among the 50–59year group in W1 (51.9%), who were aged 62–71 years in W3 (43.8%).

### Predictors of decrease in DBP and increase in awareness of hypertension

A mixed regression model that included change in DBP as the dependent variable found that the observed DBP decrease from W1 to W3 was associated with increasing age, living in rural areas, physical activity and having health insurance (Table 2). Predictors of change in prevalence of hypertension were increasing BMI and residing in rural areas (S1 Table). Female gender, ageing, residing in rural areas, increasing BMI having health insurance and diabetes predicted an increase in hypertension awareness over the two time periods (Table 3), adjusting for all variables.

**Table 2:** Multivariate analysis showing the predictors of decrease in DBP (WHO-SAGE Ghana Waves 1 and 3), n = 794.

	DBP mmHg		
Characteristics	Estimate	p-value	95% CI
*Sex*			
Male	Ref		
Female	-0.02	0.990	-3.15–3.19
Age	-0.19	< 0.001	-0.30–(-0.07)
*Location*			
Urban	Ref		
Rural	-3.63	< 0.002	-5.97–(-1.10)
*Marital Status*			
Married	Ref		
Not married	-2.36	0.933	-2.68–2.92
*Own education* (yrs)	-0.30	0.04	-0.58–(-0.01)
*Mothers education*			
Less than secondary school	Ref		
Secondary school and above	-8.06	0.059	-16.41–0.29
*Fathers Education*			
Less than secondary school	Ref		
Secondary school and above	-1.20	0.477	-2.11–4.52
*Health Insurance*			
No	Ref		
Yes	-2.60	0.037	-5.03 –(-0.16)
BMI	0.03	0.359	-0.03–0.09
*Diabetes*			
No	Ref		
Yes	0.31	0.878	-3.63–4.24
*Ever used tobacco*			
No	Ref		
Yes	0.88	0.548	-1.99–3.76
Overall physical activity	-2.65	0.030	-5.03 –(-0.25)

Note: Ref represents reference category used for the comparison. Voluntary refers to contributors to health insurance who were not captured by the insurance scheme as public or civil service workers, while mandatory refers to contributors who were employees within the public, civil and private sectors. Overall physical activity represents all activities including vigorous, moderate, walking/cycling, vigorous fitness and moderate fitness completed in a typical week. Multivariate regression was adjusted for age, sex, marital status, years of education, mother’s education, father’s education, health insurance, diabetes and overall physical activity.

**Table 3:** Odds ratio showing the predictors of change in hypertension awareness (WHO-SAGE Ghana Waves 1 and 3), n = 369.

	Hypertension Awareness		
Characteristic	OR (95% CI)	p-value	95% CI
*Sex*			
Male	Ref		
Female	1.98	0.021	1.10–3.50
Age	1.05	0.000	1.03–1.08
*Location*			
Urban	Ref		
Rural	0.58	0.017	0.37–0.91
*Marital status*			
Married	Ref		
Not married	0.92	0.753	0.54–1.56
Own years of education	1.01	0.589	0.96–1.10
*Mothers education*			
Less than secondary school	Ref		
Secondary school and above	3.65	0.053	0.98–13.54
*Fathers education*			
Less than secondary school	Ref		
Secondary school and above	0.6	0.644	0.63–4.32
*Health Insurance*			
No	Ref		
Yes	1.99	0.008	1.19–3.33
BMI	1.05	0.002	1.02–1.09
*Diabetes*			
No	Ref		
Yes	7.29	0.000	3.19–16.65
Overall physical activity	1.44	0.109	0.92–2.25

Note: Ref represents reference category used for the comparison. Voluntary refers to contributors to health insurance who were not captured by the insurance scheme as public or civil service workers, while mandatory refers to contributors who were employees within the public, civil and private sectors. Overall physical activity represents all activities including vigorous, moderate, walking/cycling, vigorous fitness and moderate fitness completed in a typical week. Logistic regression was adjusted for age, sex, marital status, years of education, mother’s education, father’s education, health insurance, diabetes and overall physical activity.

## Discussion

The main finding from this 12 year follow of older Ghanaians is an observed reduction in DBP accompanied by a reduction in hypertension prevalence. While no differences were recorded in SBP, there was a significant decrease DBP, accompanied by an increase in PP. DBP decreased for every age decile, with those aged 60–69 years at baseline having the greatest reduction over the period. A rise in hypertension awareness over the time was expected since older people are generally higher users of health services. Over the past decade, there has been an increased focus on hypertension screening activities [41] as well as heightened efforts from the health care system in Ghana to implement strategies to address NCDS [42–44]. It was not surprising that hypertension was higher among women than men due to the age of the cohort (50y+).

A lack of change in SBP between the two time periods is in contrast with many other studies that have reported a rise [17, 19, 20]. This may however be explained by the age of the cohort in W3 (median, 71years), and life expectancy in Ghana (64years) [45]. Due to the median age of the cohort, survival bias could not be ruled out, however further investigation in this regard could not be conducted due to lack of access to information on cause and time of death. The observed decline in DBP with advancing age in this older cohort is in agreement with findings from other studies. A review on the epidemiology of hypertension indicated that in most populations, DBP remained stable or reduced after age 50–60yrs [20]. Similarly, data from the Framingham Heart Study, which followed participants for three decades, showed that DBP varied with ageing, increasing until the fifth decade and gradually declining from age 60yrs onwards [19]. These age-related phenomena are explained by physiological changes in arterial structure and function. Aging is associated with an increased thickness of the arterial wall, nearly 3-fold between the ages of 20y and 90y, even in the absence of atherosclerotic plaques [46, 47]. An increase in the thickness of the arterial wall is accompanied by an increase in arterial stiffness [48], due to structural and molecular changes in the arteries (decreased elastin content, increased collagen 1 deposition, and calcification—a process termed ‘hardening of arteries’) resulting in a decline in arterial elasticity [49], which in turn induces a decline in DBP [50–52]. Due to the fall in DBP and without any change in SBP in the current study, a reduction in hypertension prevalence was observed. However, this reduction should not be misconstrued to indicate a reduction in cardiovascular risk among the cohort. A longitudinal study that evaluated the risk of cardiovascular mortality emphasized that participants whose DBP decreased with unchanged SBP had a higher cardiovascular mortality compared to those whose SBP and DBP remained constant over time. [53]. Similarly, Witteman et al. reported that, among females, a decline in DBP during a nine-year follow-up was associated with an enhancement of atherosclerotic lesions of the aorta, suggesting that a decline in DBP was a marker for atherosclerosis progression [54], reflecting prevailing compromise of the coronary circulation [50]. Hypertension is a known risk factor for atherosclerosis [55, 56]. Additionally, PP increased significantly due to the declining DBP in the current study, a situation that has the tendency to cause extensive damage to essential organs such as the brain and kidneys [49]. A large PP is associated with a greater risk for myocardial infarction, increased coronary disease and stroke even after effective BP control [22, 50, 57]. PP remains the most powerful independent predictor of cardiovascular risk in the elderly [17, 58].

The observed rise in hypertension awareness may reflect the older age of the participants who have experienced more engagement with health services over time, including routine measurement of BP at clinic visits. Other studies that included largely older participants from Ghana, China, India and Mexico reported relatively higher hypertension awareness figures [25], compared to whole-of-population studies [59]. Despite the influence of advancing age, increasing hypertension awareness in the Ghanaian population could also be attributed to several health interventions and programmes that have been implemented by the Ghana Health Service (GHS) over the past 15–20 years. The government’s broader development plan was focused on Ghana achieving middle income country status by the year 2015, through various policies, such as the Ghana Poverty Reduction Strategies (2003–2005; 2006–2009); as well as those driven by the United Nations’ Millennium Development Goals, including the Ghana Shared Growth and Development Agenda, 2010–2013 [43, 60–62]. Concurrent policies with varied mandates were also operationalized such as provision of community-based health planning and services (CHPs), which provided health information services, preventive and curative care for chronic health conditions [24, 63] and increased access to primary health care for particularly hard-to-reach communities [64]. In addition, the roll out of the national health insurance scheme in 2003 provided a major boost for services offered by CHPs and increased access to healthcare services in general, as clients were no longer required to provide out of pocket payments for some rendered healthcare services [42]. It is noteworthy that the current study reports of a dramatic increase in voluntary health insurance between W1 and W3.

Along with CHPs, the Ministry of Health, in 2005, introduced the regenerative health and nutrition programme (RHNP) as part of its effort to curb the rising trend of NCDs [65, 70] while, in 2014, the ministry collaborated with the Novartis Foundation to launch the community-based hypertension improvement program (ComHIP). This program continues to conduct community-based BP screening and BP monitoring in many parts of the country [41].

The finding that more women were hypertensive than men was expected due to the biological changes that take place in women after menopause. Estrogen has been cited to cause endothelial vasodilation, inhibit sympathetic and renin-angiotensin system (RAS) activity, enhance the production of endothelin, reduce oxidative stress, increase antioxidant production and reduce inflammation—all of which regulate BP [66–68]. After menopause, estrogen levels fall causing an increase in hypertension [69–71].

The current study found that increasing age, residing in rural areas, increasing physical activity and having health insurance were associated with a reduction in DBP between W1 and W3. The assertion that BP increases with age has been established [72, 73], however age-related BP may differ according to individual circumstances. Our finding that living in a rural setting was associated with a decline in DBP is consistent with other studies conducted in Ghana. Data from the Ghana Demographic and Health Survey 2014 which surveyed 13,265 participants nationwide reported lower BP levels in rural compared to urban areas [74]. Similarly, sub-population studies conducted in Ghana have reported lower BP in rural residents compared to their urban counterparts [75, 76]. However, contrasting findings have been reported in other recent African-based comparison studies, indicating a rising BP in rural settings [77–79]. The finding that increasing physical activity is associated with BP reduction is well known [80, 81]. In terms of dose-relationship, engagement in more regular and frequent physical activity results in greater reductions in BP irrespective of age, sex or ethnicity [82, 83].

Increased awareness of hypertension was associated with ageing, female gender, higher BMI, diabetes and having health insurance in the current study. Women were about 2 times more likely than men to be aware of their hypertensive status in the current study. This may be expected as women are known to generally have more interaction with clinics and hospitals through maternal and child health programmes and are known to place more importance on healthcare than men [84]. Furthermore, the sociocultural context (where men who sought healthcare were seen as feminine, vulnerable or weak) [85, 86] may be a disincentive for health-seeking behaviours in men. Increasing BMI predicted hypertension awareness.

This association can be attributed to the higher incidence of hypertension among obese individuals compared to those with ‘healthy weight’ [87, 88]. Increasing body weight is a growing concern in Ghana [89] and a major modifiable cardiovascular risk factor [27]. Those having diabetes were about 7 times more likely to be aware of their hypertensive status than non-diabetics in the current study. Similarly odds of hypertension awareness among diabetics have been reported [27, 90]. Hypertension is a known risk factor for diabetes [91] and the two conditions mostly coexist [92]. As such, Ghana has introduced clinics in open food markets specifically for diabetes [93] and which simultaneously offers opportunity for BP monitoring.

It was not surprising that having health insurance was associated with a decrease in DBP and an increase in hypertension awareness in the current study. The National Health Insurance Scheme (NHIS) which provides universal access to health care, has over the years improved in its efficiency and increased the range of healthcare services it covers, particularly among those of the lowest socioeconomic status and those most vulnerable groups in the population [94]. The number of outpatient visits increased sharply and health-seeking behaviour was enhanced as a result of the NHIS roll out [95, 96]. As of 2014, the NHIS had 40% coverage of Ghana’s population, an increment of 6.3% from 2010 [97, 98]. The NHIS’s widened access to most healthcare facilities in Ghana may have been an enabling factor in increase in hypertension awareness.

A major strength of the current study was the prospective design that allowed follow up of the same participants in two large countrywide studies of persons aged 50 years and older using the same study design and methodology. Validity of data was achieved using extensively trained teams of interviewers that used standard protocol and instruments and inherent quality control measures. Limitations were the high rate of loss to follow up and the inability to generalize the findings over the entire population because of the higher age of the cohort and lack of weighting applied.

## Conclusions

Despite a reduction in DBP recorded between WHO-SAGE Ghana W1 and W3 (2007–2019), hypertension prevalence in W3 remained high. An increase in awareness of hypertension over this time period suggests improved health services for BP monitoring, but this was not accompanied by an increase in the proportion of hypertensive participants who were receiving treatment for the condition, nor improved BP control. Factors associated with a reduction in DBP included increasing age, residing in a rural area and having health insurance, while predictors of hypertension awareness included higher BMI and having voluntary health insurance. This data suggests that existing health programmes should be intensified to improve the management of hypertension in Ghana.

## Supporting information

S1 Table Odds ratio showing the predictors of decrease in hypertension prevalence (WHO-SAGE Ghana Waves 1 and 3), n = 368. (DOCX)

S2 Table Characteristics of Wave 1 participants included in the study (50+y, n = 820) and those excluded (n = 3,904). (DOCX)

S3 Table Characteristics of Wave 1 participants included in the study (50+y, n = 820) and those followed-up but excluded from analysis (n = 79). (DOCX)
