# Supplementary material for: Determinants of change in blood pressure in Ghana: Longitudinal data from WHO-SAGE Waves 1–3
Source: PLoS One. 2021 Jan 8;16(1):e0244807. doi: 10.1371/journal.pone.0244807 (PMC7793275; doi:10.1371/journal.pone.0244807)
Supplement: S1 Table — (DOCX) [file pone.0244807.s001.docx]

S1 Table: Odds ratio showing the predictors of decrease in hypertension prevalence (WHO-SAGE Ghana Waves 1 and 3), n = 368

| Hypertension prevalence | | | |
| --- | --- | --- | --- |
| Characteristic | OR  (95% CI) | p-value | 95 CI |
| *Sex*  Male | Ref |  |  |
| Female | 1.08 | 0.759 | 0.66-1.76 |
| Age | 0.99 | 0.422 | 0.98-1.01 |
| *Location*  Urban | Ref |  |  |
| Rural | 0.48 | < 0.01 | 0.30-0.77 |
| *Marital Status*  Married | Ref |  |  |
| Not married | 1.80 | 0.125 | 0.45-1.10 |
| *Own years of education* | 1.04 | 0.076 | 0.99-1.09 |
| *Mothers education*  Less than secondary school | Ref |  |  |
| Secondary school and above | 0.64 | 0.397 | 0.23-1.78 |
| *Fathers education*  Less than secondary school | Ref |  |  |
| Secondary school and above | 0.91 | 0.727 | 0.55-1.52 |
| *Health Insurance*  No | Ref |  |  |
| Yes | 1.33 | 0.138 | 0.91-1.01 |
|  |  |  |  |
|  |  |  |  |
| BMI | 0.98 | 0.135 | 0.96-1.01 |
| *Ever used alcohol* |  |  |  |
| No | Ref |  |  |
| Yes | 0.97 | 0.885 | 0.67-1.40 |
| *Diabetes* |  |  |  |
| No | Ref |  |  |
| Yes | 0.57 | 0.122 | 0.27-1.16 |
| *Ever used tobacco* |  |  |  |
| No | Ref |  |  |
| Yes | 0.78 | 0.284 | 0.49-1.22 |
| Overall physical activity | 1.04 | 0.821 | 0.71-1.53 |
|  |  |  |  |

Note: Ref represents reference category used for the comparison. Voluntary refers to contributors to health insurance who were not captured by the insurance scheme as public or civil service workers, while mandatory refers to contributors who were employees within the public, civil and private sectors. Overall physical activity represents all activities including vigorous, moderate, walking/cycling, vigorous fitness and moderate fitness completed in a typical week. Multivariate regression was adjusted age, sex, marital status, years of education, mother’s education, father’s education, health insurance, diabetes and overall physical activity.
