# Supplementary material for: Determinants of change in blood pressure in Ghana: Longitudinal data from WHO-SAGE Waves 1–3
Source: PLoS One. 2021 Jan 8;16(1):e0244807. doi: 10.1371/journal.pone.0244807 (PMC7793275; doi:10.1371/journal.pone.0244807)
Supplement: S2 Table — (DOCX) [file pone.0244807.s002.docx]

S2 Table: Characteristics of Wave 1 participants included in the study (50+y, n = 820) and those excluded (n = 3,904)

| Characteristic | Wave 1  included | Wave 1  excluded | p-value |
| --- | --- | --- | --- |
| Age in years, Median (IQR) | n = 820  59 (13) | n = 3904  64 (17) | <.0001 |
| Location: Urban, n(%) | n = 820  364 (44.4) | n = 3904  1561 (40.0) | 0.0196 |
| Gender: Male, n(%) | n = 820  433 (52.8) | n = 3904  1920 (49.2) | 0.2080 |
| Education: Schooled, n (%) | n = 817  398 (48.7) | n = 3476  1539 (44.3) | 0.0217 |
| Marital status:  Married/ cohabiting, n (%) | n = 817  489 (59.9) | n = 3880  2250 (58.0) | 0.3262 |
| SR Hypertension, n (%) | n = 820  95 (11.6) | n = 3469  490 (14.1) | 0.0567 |
| SR Diabetes, n (%) | n = 820  17 (2.1) | n = 3469  150 (4.3) | 0.0027 |
| SR Depression, n (%) | n = 820  13 (1.6) | n = 3469  54 (1.6) | 0. 9524 |
| SR Health, very good n (%) | n = 820 | n = 3481 | 0.1893 |
|  | 39 (4.8) | 131 (3.8) |  |
