# Supplementary material for: Determinants of change in blood pressure in Ghana: Longitudinal data from WHO-SAGE Waves 1–3
Source: PLoS One. 2021 Jan 8;16(1):e0244807. doi: 10.1371/journal.pone.0244807 (PMC7793275; doi:10.1371/journal.pone.0244807)
Supplement: S3 Table — (DOCX) [file pone.0244807.s003.docx]

**S3 Table: Characteristics of Wave 1 participants included in the study (50+y, n = 820) and those followed-up but excluded from analysis (n = 79)**

| Characteristic | Wave 1  included | Wave 1 followed-up but excluded | p-value |
| --- | --- | --- | --- |
| Age in years, Median (IQR) | n = 820  59 (13) | n = 79  59 (13) | 0.7791 |
| Location: Urban, n(%) | n = 820  364 (44.4) | n = 79  30 (38.0) | 0.2724 |
| Gender: Male, n(%) | n = 820  433 (52.8) | n = 79  31 (39.2) | 0.0210 |
| Education: Schooled, n (%) | n = 818  398 (48.7) | n = 25  11 (44.0) | 0.6422 |
| Marital status:  Married/ cohabiting, n (%) | n = 817  489 (59.9) | n = 77  60 (77.9) | 0.0018 |
| SR Hypertension, n (%) | n = 820  95 (11.6) | n = 24  3 (12.5) | 0.7520 |
| SR Diabetes, n (%) | n = 820  17 (2.1) | n = 24  1 (4.2) | 0.4082 |
| SR Depression, n (%) | n = 820  13 (1.6) | n = 24  0 (0.0) | - |
| SR Health, very good n (%) | n = 820 | n = 25 | 1.0000 |
|  | 39 (4.8) | 1 (4.0) |  |
